# Multimodality Imaging of Acute Myocarditis in Cytokine Release Syndrome (CRS) Following CAR-T Therapy

**DOI:** 10.1016/j.cjco.2024.04.004

**Published:** 2024-04-17

**Authors:** Janane Maheswaran, Moezedin Javad Rafiee, Sydney Cordes, Benedicte Lefebvre, Michael Chetrit

**Affiliations:** a Division of Cardiology, McGill University, Montreal, Quebec, Canada; b Division of Diagnostic Radiology, McGill University, Montreal, Quebec, Canada; c Wilfrid Laurier University, Waterloo, Ontario, Canada; d Division of Cardiology, University of Pennsylvania, Philadelphia, Pennsylvania, USA

**Chimeric antigen receptor (CAR T)-cell therapy is a novel immunotherapy with promising results for patients with advanced malignancies.** **^1^** **Cytokine release syndrome is an adverse effect of this therapy, associated with multiorgan, including cardiovascular, complications.** **^1^** **We describe a case of CAR T-cell–associated cardiotoxicity and the associated multimodality imaging findings.**

## Case Description

A 74-year-old woman known for hypertension, hypothyroidism, psoriasis, fibromyalgia, and internal jugular vein thrombosis was admitted for CAR T-cell therapy due to relapsed and refractory diffuse large B-cell lymphoma (DLBCL), which was diagnosed 14 months previously, when she presented with flank pain and fatigue.

Laboratory evaluation at that time was notable for anemia and thrombocytopenia. An initial abdominal computed tomography (CT) scan revealed multiple enlarged, conglomerated intra-abdominal lymph nodes. Subsequent 18F-fluorodeoxyglucose (FDG)–positron-emission tomography and CT showed diffusely enlarged, 18F-fluorodeoxyglucose–avid lymph nodes in the abdomen and thorax. A surgical lymph node biopsy confirmed the diagnosis of diffuse large B-cell lymphoma. She received 5 courses of treatment with rituximab, cyclophosphamide, doxorubicin, vincristine, and prednisone (R-CHOP), which was completed 7 months prior to her current admission, with no significant complication.

A follow-up positron-emission tomography and CT scan done 5 months prior to the current admission showed disease progression. A repeated biopsy from cervical lymph nodes confirmed a relapse of DLBCL (CD20 +, CD 10 –). A bone marrow biopsy showed no sign of lymphoma.

Considering her refractory and/or relapsed DLBCL, the decision was made to administer CAR T-cell therapy. Her baseline cardiac workup done 6 months after she received 5 courses of R-CHOP treatment, but prior to starting CAR T-cell therapy, demonstrated a normal left ventricular ejection fraction (LVEF) of 62%, with no regional wall-motion abnormality. Baseline inflammatory markers were elevated with a C-reactive protein (CRP) level of 22.6 mg/L (normal range: 0-5.00 mg/L), and a ferritin level of 707 mg/L (normal range: 11.0-306.0 mg/L), reflecting a baseline inflammatory state. No clinical signs of infections or other concurrent systemic processes were present, and the patient received 4 doses of COVID-19 vaccination, with the fourth dose being given 15 months prior to her CAR T-cell therapy; therefore, her baseline inflammatory profile was attributed to the underlying malignancy.

She underwent lympho-depletion with fludarabine (30 mg/m^2^) and cyclophosphamide (500 mg/m^2^) for 3 days, starting 5 days prior to CAR T-cell infusion, and she subsequently received an infusion of YESCARTA (axicabtagene ciloleucel; Kite Pharma Inc) CAR T-cells. Her baseline complete blood counts prior to receiving CAR T-cell therapy were as follows: white blood cells, 500 (4500-11,0000); hemoglobin: 75 g/L (120-160); and platelets 94,0000 (140,0000-450,0000). On day 1 post–CAR T-cell infusion, she developed low-grade cytokine release syndrome (CRS) according to the American Society for Transplantation and Cellular Therapy grading system for CRS,^1^ characterized by fatigue, low-grade fever (38.5 degree), and palpitations. She denied chest pain and shortness of breath. A resting electrocardiogram confirmed sinus tachycardia with a heart rate of 124 beats per minute. On day 5 post–CAR T-cell infusion, she continued to be febrile and showed signs of respiratory distress, and had mild hypoxia and significant tachycardia up to 160 beats per minute consistent with grade-2 CRS. A chest radiograph revealed pulmonary edema. Lab tests revealed the following: mild anemia with a hemoglobin of 93 g/L (normal range: 120-160); thrombocytopenia with a platelet count of 93 10ˆ9/L (normal range: 140-450); a white blood cell count of 4.9 10ˆ9/L (normal range: 4.5-11); an elevated C-reactive protein level of 113 mg/L (normal range: 0-5.00); a ferritin level of 3473 ug/L (normal range: 11.0-306.0); an N-terminal pro-B-type natriuretic peptide level of 11,259 pg/mL (normal: < 125); and an Hs troponin I level of 2291 ng/L (normal: < 12.0), raising the suspicion of an inflammatory process with myocardial involvement. She received nasal oxygen and was empirically started on broad-spectrum antimicrobial therapy once blood cultures were obtained.

**Figure 1:**
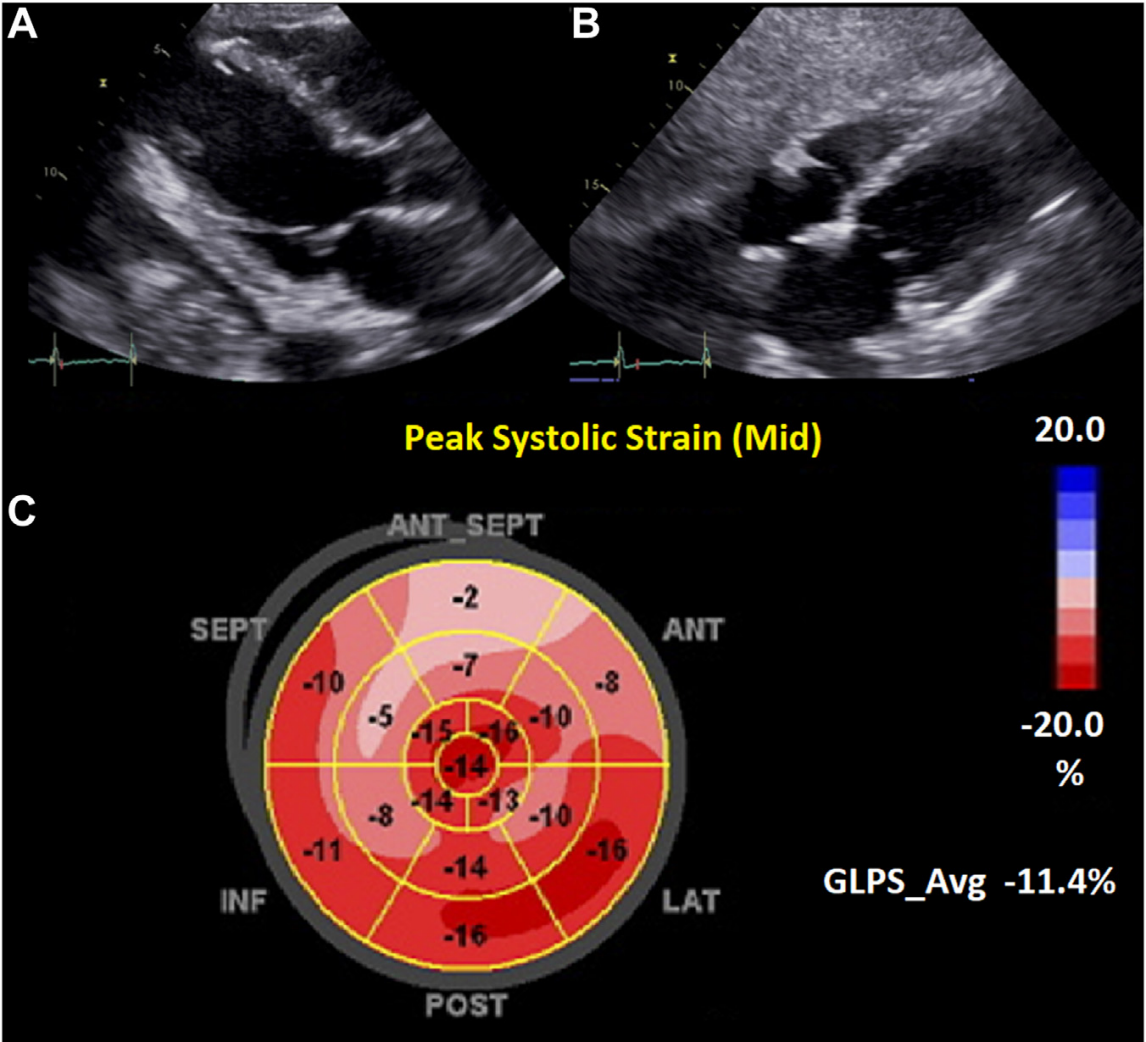
Transthoracic echocardiogram images in patient with chimeric antigen receptor CAR T-cell–induced myocarditis. (**A**) Parasternal long-axis view; (**B**) subcostal view showing no significant pericardial effusion; and (**C**) left ventricular strain demonstrating overall low global strain of –11.4%, and regional abnormalities of the basal to mid anterior and anteroseptum (ANT_SEPT). ANT, anterior; GLPS, Global Longitudinal Peak Strain; HR, heart rate; INF, inferior; LAT, lateral; LAX, POST, posterior; SEPT, septal.

She received intravenous diuretic therapy and was transferred to the intensive care unit for further monitoring. A transthoracic echocardiogram was done upon intensive care unit admission, which showed a severely reduced LVEF of 25%-30%, an abnormal global peak longitudinal strain at –11.4%, and regional wall-motion abnormalities most notable for akinesia of the mid-inferoseptal segment, severe hypokinesia of the mid and apical inferior and anteroseptal segments, and mild–moderate hypokinesia of the other segments (Fig. 1). CART-cell–induced cardiotoxicity was suspected in view of the temporal correlation of CAR T-cell–therapy administration and clinical deterioration. No signs of systemic infection were present, and lab work was not suggestive of tumour lysis syndrome. Per hospital guidelines for the management of CAR T-cell–associated toxicity, she received immunosuppressive therapy with both tocilizumab (an anti-interleukin-6 antibody) and corticosteroids, with subsequent clinical improvement, with no signs or laboratory parameters to suggest cardiogenic shock, and she had down-trending Hs troponin I levels, from 2291 ng/L to 38.8 ng/L. She was weaned off supplemental oxygen with diuretics alone and did not require further escalation of therapy with inotropes.

**Figure 2:**
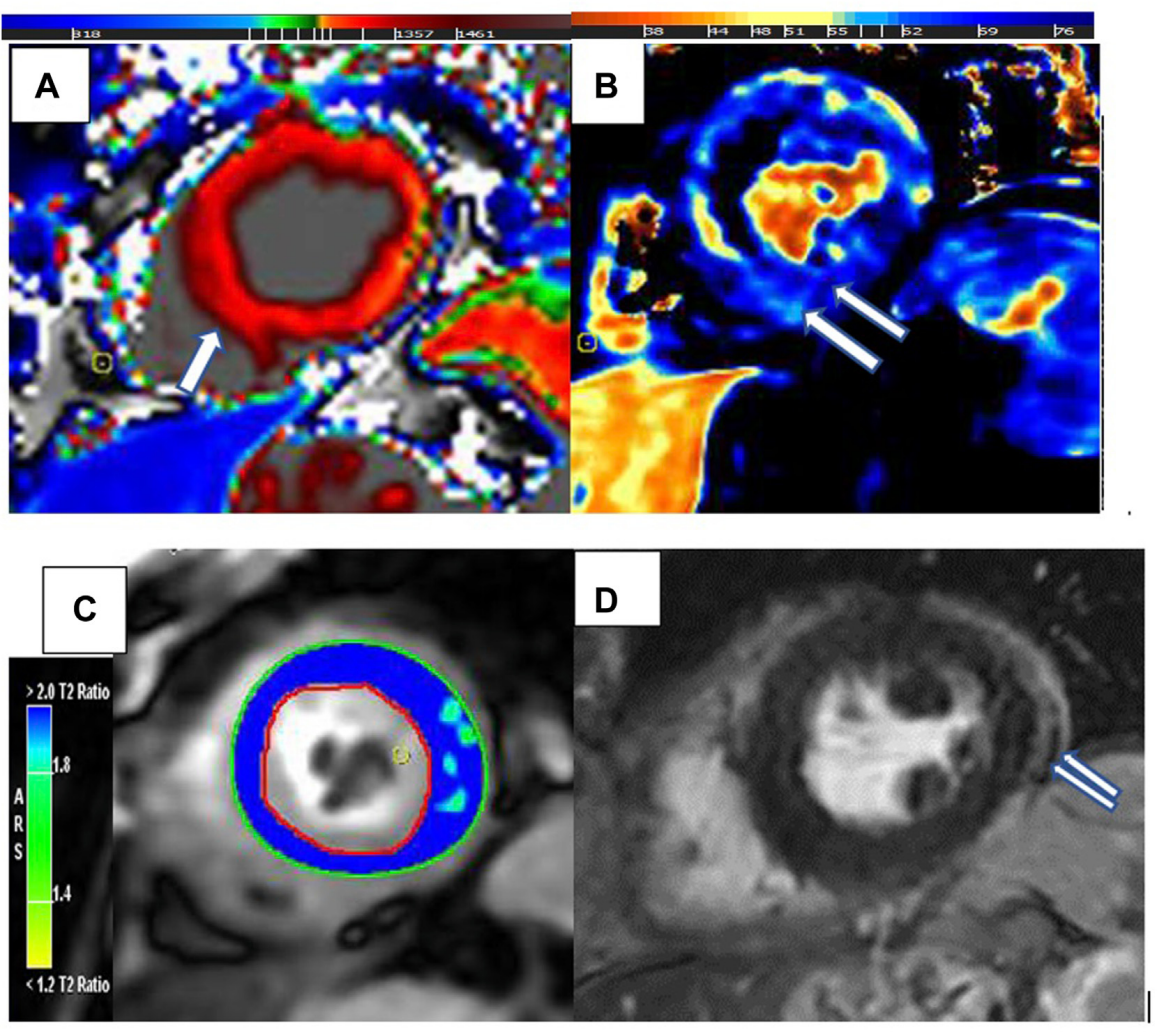
(**A**) T1 mapping of the short-axis mid-ventricular slice of the left ventricle (LV) demonstrating diffusely prolonged T1 relaxation time (**red**) with very minimal normal relaxivity along the lateral wall (**green**). Increased T1 values in noted globally (**arrow**). (**B**) T2 mapping of the short-axis mid-ventricular slice of the LV demonstrating diffusely prolonged T2 decay (**blue**) with very minimal normal decay in the mid-septum and anterolateral walls (**yellow**). Increased T2 values globally sparing the mid septum (**arrows**). The combination of (**A**) and (**B**) is indicative of inflammation. (**C**) T2 Short Tau Inversion Recovery (STIR) sequence of the short-axis mid ventricular slice of the LV, demonstrating increased signal compared to the skeletal muscle signal (**blue**), reflecting the diffuse edema noted on the (**B**) T2 map. (**D**) Evidence of nonischemic pattern of late gadolinium enhancement manifesting as linear subepicardial enhancement of the mid entire lateral wall in short-axis view (**thin arrows**) on late gadolinium enhancement sequence associated with enhancement of the adjacent pericardium.

Given the high clinical suspicion for CART-cell–induced cardiotoxicity, on day +14 post–CAR T-cell therapy, cardiac magnetic resonance imaging (CMR; Fig. 2) was performed to evaluate myocardial injury. The CMR revealed mild global hypokinesia, diffuse myocardial edema as elevated T2-values, and increased myocardial signal intensity on T2-map and T2-weighted Short Tau Inversion Recovery (STIR) images. There was also nonischemic myocardial injury in the form of diffusely prolonged T1-relaxtaion times on T1-map and subepicardial and mid-myocardial late enhancement involving mid to distal anterior and inferolateral segments adjacent to a tiny pocket of pericardial fluid on late gadolinium enhancement (LGE) images. Left ventricular (LV) systolic function was improved significantly compared to the last echocardiography (59% vs 25%).

## Discussion

CAR T-cell therapy has emerged as a promising biologic therapy for refractory and/or relapse hematologic malignancy. CRS is the most common adverse event following CAR T-cell therapy, with an incidence rate of 85%-93%. Among these cases, 0%-46% manifest a severe or life-threatening form.^1^^,^^2^ Retrospective studies have shown that 10%-20% of patients with high-grade CRS post–CAR T-cell therapy (grades 3 and 4) experience cardiovascular complications, including cardiomyopathy, heart failure, new-onset arrhythmias, and myocardial infarction.^2^ Acute myocardial injury in the context of CRS shares similarities with sepsis-related cardiomyopathy and is thought likely to be mediated by interleukin-6, a cytokine elevated during infectious and inflammatory processes.^3^ We acknowledge that CAR T-cell–induced myocarditis might be a challenging diagnosis to make, in view of other potential confounders, such as other cardiotoxic agents. In our case, our patient was 7 months post–doxorubicin therapy, with a normal transthoracic echocardiogram prior to initiating CAR T-cell therapy, making doxorubicin cardiotoxicity less likely. Cyclophosphamide cardiotoxicity also was considered in the differential diagnosis, as it was administered for 3 days, starting 5 days prior to CAR T-cell therapy. Although a few case reports have been made of cyclophosphamide-induced cardiotoxicity, such cases typically have a poor prognosis, due to the direct damage to endothelial capillaries, leading to the leakage of proteins and erythrocytes and the development of hemorrhagic pericarditis, interstitial hemorrhage, and coronary microthrombi. According to these studies, echocardiographic findings in cyclophosphamide-related cardiotoxicity have shown significant pericardial effusion and marked myocardial thickening,^4^ which our case did not illustrate. Additionally, the timing of symptoms is more consistent with CRS. Nonetheless, the potential for a synergistic cardiotoxic effect between cyclophosphamide and CAR T-cell therapy cannot be ruled out entirely without autopsy.

Another potential cause of cardiomyopathy in our patient could be sepsis-related or septic cardiomyopathy. This condition is characterized by reversible myocardial dysfunction triggered by an immune response to an infection.^3^ Although septic cardiomyopathy has no standard definition, It is generally described as cardiac dysfunction that is unrelated to ischemia, with one or more of the following criteria: (i) a temporary reduction in LVEF to less than 50%; (ii) LV dilation under normal or low filling pressure; and (iii) right ventricular dysfunction and/or LV dysfunction with a poor response to fluid infusion.^3^ Recent research by Muehlberg et al. has shown that, using myocardial T2-mapping, myocardial edema is a common finding in patients with septic cardiomyopathy, as is acute myocarditis.^5^ Their findings suggest that the reversibility of cardiac dysfunction and the lack of subepicardial fibrosis on CMR imaging can serve to distinguish between septic cardiomyopathy and myocarditis. In our patient, the clinical improvement and the trend toward normalization of troponin levels with tocilizumab treatment, associated with the presence of nonischemic myocardial late enhancement on LGE CMR sequences, are indicative of a pattern consistent with acute myocarditis induced by CAR T-cell therapy.

Transthoracic echocardiogram is often one of the first-line imaging modalities used to evaluate baseline cardiac function, and it serves as a useful tool to do follow-up on cardiac function during the course of potentially cardiotoxic therapies. Worsening cardiomyopathy in the setting of cardiotoxic treatment has been described as a drop in LVEF of more than 10% from baseline, to less than 50%.^6^ LV global longitudinal strain (GLS) is emerging as an earlier method of detecting subclinical myocardial dysfunction. GLS has been particularly helpful in identifying individuals with an LVEF of 50%-59% who develop subclinical LV dysfunction in the setting of cardiotoxic medications, described as a GLS more positive than –16%.^7^ Important to note is that similar subclinical dysfunction can be present in the setting of chemotherapy-induced myocarditis; however, current strain-based techniques are unable to differentiate one such type of dysfunction from another. As a consequence, if clinical suspicion for myocarditis is present (including, but not limited to, regional strain dysfunction in the basal inferior and inferolateral segments, markedly elevated troponin and elevated inflammatory markers), additional imaging with CMR is often needed. Our patient demonstrated both of these findings on her echocardiogram—she had a > 10% drop in LVEF, from 62% to 25%-30%, and she had an abnormal GLS of –11.4%, in addition to elevated inflammatory markers and troponins.

CMR has been established as the noninvasive gold standard for detecting myocardial injuries across a wide range of cardiotoxic causes. CMR assesses both cardiac function and tissue characterization, including myocardial edema that is accompanied with acute myocardial injury. CMR can help identify evidence of active myocardial inflammation, by using the parametric cardiac modalities, such as T1 and T2 mapping.^2^ In this patient, the CMR shows widespread myocardial edema, as well as a combined subepicardial and mid-myocardial pattern of LGE with smooth enhancement of the pericardium, which may be secondary to the CRS. The previous case report utilized CMR to identify CAR T-cell–associated myocardial damage in patients with cardiac lymphomatous involvement or preexisting cardiomyopathy. The pattern and type of late myocardial enhancement corresponded to the prior cardiac lymphomatous involvement, suggesting past scarring. However, our case describes magnetic resonance imaging (MRI) findings in a patient with no baseline cardiac abnormalities. CMR has been recommended for use in follow-up of patients with suspected cardiac involvement post–CAR T-cell therapy, particularly those presenting with sustained sinus tachycardia, hypotension, elevated troponin, impaired LV function (LVEF < 50% or decreased > 10%), right ventricular dysfunction, and arrhythmias.^2^ In conclusion, we recommend the use of multimodality imaging with transthoracic echocardiogram and cardiac MRI during the initial evaluation and for subsequent monitoring of patients with suspected CAR T-cell therapy induced cardiotoxicity (Videos 1and2).

**Novel Teaching Points**

• A diagnosis of CAR T-cell–therapy–mediated CRS and associated cardiotoxicity should be considered in individuals who develop clinical signs and/or symptoms of heart failure while receiving this potentially life-saving therapy.
• Multimodality imaging, with the use of transthoracic echocardiogram and cardiac MRI, is recommended for further evaluation when concerns of CAR T-cell–mediated cardiotoxicity are present.

## Supplementary Material

Video 1 Parasternal long axis view (echocardiogram): Parasternal long axis view showing severe hypokinesia of the mid anteroseptal segment, moderate hypokinesia of the basal anteroseptal and mid inferolateral segments, and mild hypokinesia of the basal inferolateral segment.

Video 2 Parasternal short axis view (echocardiogram): Parasternal short axis view showing akinetic mid inferoseptal segment, severely hypokinetic mid inferior and mid anteroseptal segments, moderately hypokinetic mid anterior and mid inferolateral segments, and mildly hypokinetic mid anterolateral segment.
